# Childhood abuse as a mediator of the relationship between early family socio-economic status and geriatric depression: A population-based study in China

**DOI:** 10.1016/j.heliyon.2023.e22021

**Published:** 2023-11-07

**Authors:** Chengcheng Liu, Mingyu Zhang, Chongyue Ma, Mingqi Fu, Jing Guo, Cheng Zhen, Bo Zhang

**Affiliations:** a The School of Social Development and Public Policy, Beijing Normal University, Beijing, 100875, PR China; b School of Public Health, Peking University, Beijing, 100191, PR China; c School of Accounting, Henan University of Economics and Law, PR China; d School of Public Management, Central South University, Wuhan, 430079, PR China; e Center For the History of Medicine, School of Health Humanities, Peking University Health Science Center, Beijing, PR China; f Department of Neurology and ICCTR Biostatistics and Research Design Center, Boston Children's Hospital and Harvard Medical School, Boston, MA, USA; g Health Policy and Technology Assessment Center, Peking University Health Science Center, Beijing, PR China

**Keywords:** Childhood abuse, Psychological health, Traumatic stress theory, Mediating effect

## Abstract

Previous studies have suggested that childhood socioeconomic status (SES) is linked to geriatric depressive symptoms in many developed countries. However, the potential pathways of the relationship between childhood SES and geriatric depressive symptoms need to be further explored. This study aimed to assess the mediating effect of being abused during childhood on the association between childhood SES and geriatric depressive symptoms, using evidence from a longitudinal study in China. The study cohort included 8137 individuals. Childhood abuse was defined as experiences related to parental violence, sibling abuse, school violence, community violence, and parental quarrel. Results indicated poor childhood SES was associated significantly with geriatric depressive symptoms. The indirect effect of poor childhood SES to high geriatric depressive risk through community violence, sibling abuse, school violence, and parental quarrel were 0.02, 0.01, 0.02, and 0.01, respectively. Our findings shed new light on the literature regarding the impact of childhood SES on elderly depressive symptoms. Furthermore, childhood SES demonstrated a significant correlation with geriatric depressive symptoms through bullying behaviors. The findings highlight the need to promote both childhood social welfare and psychological well-being within the elderly population.

## 1 Introduction

Depression is a prevalent health issue, afflicting a huge number of individuals globally. Studies have found that depression risk grows along with age [1], and is particularly high among older adults [2]. While literature published in the late 20th century suggested that major depression occurs in 1 %–3 % of the general elderly population [3,4], recent studies pointed out a more serious situation. In Ghana, the prevalence of depressive symptoms among people over 65 years old was as high as 37.8 % [5]. Japan, a fast-paced society with an aging population, suffered severely from mental disease also, with about 1.3 million elder citizens reported to have severe depressive symptoms in 2017, which represents an increase of 38 % from 2005 [6]. A roughly equal prevalence occurred in Korea, where around 21 % of older adults were identified as patients with severe depressive symptoms. China, the country with the greatest number of senior citizens, also faces enormous challenges from elderly depressive symptoms. According to a meta-analysis conducted in the context of Chinese society, the incidence of geriatric depressive symptoms was 20.0 % [7]. Along with its high prevalence, depression in the elderly is an important public health problem causing considerable morbidity, disability or even suicidality [8,9]. Yet, limited medical treatment is received by the elderly due to economic problems, self-neglect and other issues [10,11]. Therefore, it is reasonable to urge that more attention be paid to the elderly afflicted by depressive symptoms.

Various helpful and essential risk factors analyses have been conducted regarding understanding and managing geriatric depressive symptoms. Among the proposed risk factors, socioeconomic status (SES) is mentioned frequently. Scientific research indicates that exposure to conditions typical of socioeconomic status (SES) during childhood exhibits a correlation with children's health, cognitive, and socioemotional consequences [12,13]. The impact commences even before birth and endures until adulthood. Many studies have been designed to investigate the relationship between SES and depressive symptoms [14,15], suggesting that disadvantaged people are more likely to develop depression [16]. A review of previous literature finds that the association between SES and depressive symptoms is mainly discussed through three plausible channels: (i). Disadvantaged SES leads to life stresses directly, which have the potential to be deleterious to mental health [17]. (ii). SES impacts an individual's sense of self-actualization and safety. People with high SES will have less anxiety and stress when coping with traumatic events which prevents them from evolving into depression [18]. (iii). SES influences lifestyle [19]. People with high SES are more likely to have a lifestyle that includes protective factors, such as extensive social support and more leisure time. Therefore, researchers currently agree that lower SES is a risk factor associated with higher depressive symptoms.

Furthermore, because a relatively long time is needed for the three mentioned channels to come into being, some studies seek to explain the association between SES and the level of geriatric depressive symptoms within a life-long framework. According to the Life Course Approach, earlier conditions and events have long-term effects on people's later lives, including their health status [20]. Extending this insight, this study hypothesized that the trajectory started in childhood has important implications for mental health status in adulthood. However, whether childhood SES has a long-lasting effect on later geriatric depressive symptoms is still unclear. While one study conducted in Japan validated the influence of childhood SES on elderly depression [21], no significant and independent association was found in New Zealand [22]. In some studies, the elderly were believed to be more prone to depressive symptoms because they are less able than the young to exclude unwanted experiences, such as economic hardship in childhood [23]. On the contrary, other studies proposed that the influences brought by earlier life experiences would be replaced by later events, explaining why the magnitude of the association between financial hardship and the onset of mental disorders decreases with age despite being significant at all life stages (including childhood, adolescence, and adulthood) [24]. Therefore, the association between childhood SES and geriatric depressive symptoms has never been without controversy in different social settings and has not been fully investigated in the context of Chinese society. Further study to examine this association is needed.

Although many theories are proposed to understand the relationship between childhood SES and geriatric depressive symptoms, in this study we used the traumatic stress theory as guidance. Traumatic stress theory has noted that children of lower SES backgrounds would be at greater risk of experiencing traumatic stress because of the vulnerable features developed by marginalized groups [25]. As the prior investigation has documented, children growing up in lower SES families have a higher risk of being exposed to parental violence than children with higher SES [26]. Meanwhile, children residing in disadvantaged neighborhoods were also viewed as the result of lower SES and more peer abuse/violence in those disadvantaged neighborhoods [27]. For example, a survey of 3437 families conducted in Spain found more violent behaviors in neighborhoods with greater deprivation [28]. Thus, there are multiple risk factors for bullying embedded in children's broader socio-environment contexts, including family and neighborhood (i.e. school, and community) contexts [29]. The combined effect of these traumatic experiences would deteriorate people's mental health status. A growing body of research has provided support for the passive outcomes of the mental health perspective brought by abuse at earlier life stages [30]. Thus, it is reasonable for this study to assume that being abused during childhood may mediate the relationship between childhood SES and elderly depressive symptoms. If this hypothesis were proven, results would be helpful to guide prevention and intervention efforts and reduce disparities in bullying risks, especially in children from low SES families.

The relationship between childhood SES and geriatric depressive symptoms has been the subject of numerous studies, but it has never been without controversy in various social contexts and hasn't been well examined in the context of Chinese society. Therefore, further study to examine this association is needed. In addition, there have been many theories proposed to understand the relationship between childhood SES and geriatric depressive symptoms, however, the traumatic stress theory may offer a fresh and important explanation. In this work, we explored a potential road to geriatric depression using the four different forms of childhood abuse as mediators and the traumatic stress theory as a guide. Using data from the China Health and Retirement Longitudinal Study (CHARLS), the aim of this study was to assess the mediating effect of being abused during childhood within the association between childhood SES and geriatric depressive symptoms.

The structure of the current study is as follows. The backgrounds of the research problem and research objectives are explained in Section 1. It also reviews relative literatures, highlights the research gap, and discusses the underlying theoretical concepts of variables considered for the conceptual framework. In Section 2, which covers the methodology of the study, it is stated how the variables were measured and how the statistical analysis was done. The results of this investigation are described in Section 4. The debate is expanded in Section 5 based on the study's findings, and pertinent interventions and policies are suggested. Section 6 identifies limitations and further studies. Section 7 summarizes this study, and then provides the brief findings, limitations, and further studies.

## 2 Methods

### 2.1 Study design

This study is a secondary analysis of de-identical public data from the China Health and Retirement Longitudinal Study (CHARLS). CHARLS is a national representative survey conducted among Chinese aged 45 years and over, designed to reveal the dynamics of retirement in China and find out how retirement interacts with health and individuals' well-being. CHARLS had its baseline survey in 2011, with exams performed every 2 years for a total of 4 waves from 2011 to 2018. An additional life history survey was conducted in 2014 to document CHARLS respondents’ important life events, such as neighborhood quality and SES during childhood. Trained interviewers conducted face-to-face interviews with selected respondents, and all voluntary participants gave written informed consent after being informed of the aim of the survey and the right of refusal. The current study used open-access data from the life history survey in 2014 and the survey in 2015.

The CHARLS selected individuals through four-stage, stratified, cluster sampling. In the first stage, 150 counties from 28 provinces were randomly selected to demonstrate the socioeconomic and geographic patterns in modern China. In the second stage, three primary sampling units were chosen within each county through probability proportion to their population size. Then, with dwellings in each selected primary sampling unit outlined on maps, a sample of 24 households was randomly selected. Finally, one member aged 45 years or older in each household was randomly chosen as a respondent in this survey. For those who were deceased or missing during follow-up, a new sample was selected for replacement. This study used follow-up data in 2014 (Life History Survey) and 2015 with a matched ID number, a total of 18628 respondents answered both surveys. In this study, we limited our sample to the elderly who answered the questions about depressive symptoms, and 8137 individuals were included.

### 2.2 Measurements

#### 2.2.1 Major dependent variables

**Depressive symptoms** in this study were measured by the 10-item Center for Epidemiological Studies Depression Scale (CESD) [31]. This scale has shown high reliability and validity [32], and has been widely used to measure depressive symptoms among Chinese older adults [8,33]. Participants reported the frequency they experienced depressive symptoms (respectively as 10 items of the CESD) during the last week before the interview, with responses ranked between 0 and 3 [0 = Rarely or none of the time (less than 1 day); 1 = some or a little of the time (1–2 days); 2 = Much or a moderate amount of the time (3–4 days); 3 = Most or all of the time (5–7 days)] [34]. The total score ranks from 0 to 30, with higher scores indicating a higher level of depressive symptoms. Scored 10 or above indicated significant depressive symptoms in this study [31], and Cronbach's alpha for the ten items was 0.80.

#### 2.2.2 Major independent variables

**Childhood Socioeconomic Status** (SES) was correlated with household income and financial [35]. Referring to previous studies, childhood SES was measured by asking participants to indicate their perceived social class in childhood [36,37]. In this study, it was determined by the question: “Compared with the average level in your community, how do you perceive your family's financial situation when you were a child before age 17?” The answer to this question ranged from 1 (much better) to 5 (much worse), a higher score indicating a worse SES.

**Childhood abuse**. Previous study regarding abuse has emphasized the association between individuals and their immediate environment [38]. Especially for children, bullying has been documented as a phenomenon taking place in several socio-environmental contexts, including school, home and community [39,40]. Based on the socio-environment of children, the current study adopted several types of child abuse embedded in family, community or school to examine children's abuse experiences, including parental violence, parental quarrel, sibling abuse, school violence, and community violence. All those types of child abuse are viewed as critical forms of child abuse in previous studies [41].

#### 2.2.3 Potential confounding variables

**Socio-demographic variables** of the respondents included in this study were gender (male/female), marital status (have spouse/without a spouse), age, income, education, residence (rural areas/urban areas), nationality (Han/minority). Self-reported health status (better/poor) was also included in the analysis based on previous studies [42].

**Some adverse experiences** were measured in the current study after considering that the influences brought by earlier life experiences would be replaced by later events [24]. Previous studies found a strong correlation between marital quality and mental health status, with positive marital quality might enhance mental health and mental health might be undermined by negative marital quality [43]. In the current study, two questions were used to measure responders’ marital quality: 1) Was it a voluntary marriage when you got married to your spouse? 2) Did you stop living with your spouse during adulthood? Yes or No are the options for those two questions. Physical health is another factor mentioned frequently regarding the studies of mental health status, with disability issues and chronic diseases are two important signs of poor physical health, which were both pointed as risk factors for depressive symptoms in the previous studies [44,45]. Thus, disability issues (Not have disabilities issues/Have disabilities issues during childhood/Have disabilities issues during adulthood), chronic diseases (Not have chronic diseases/Have chronic diseases during childhood/Have chronic diseases during adulthood) were included in our study. Furthermore, the current study used parental death (Not have/Either father or mother dies during childhood/Either father or mother died during adulthood) as a critical adverse experience since the loss of a parent is one of the most stressful events that a person can experience, individuals who were parentally bereaved tend to be at greater risk for depressive symptoms [46].

**Parents’ mental health status** (with depressive symptoms or without) was also included in this study, evaluated by the question “During the years you were growing up, had your male/female guardian showed continued signs of sadness or depression that lasted 2 weeks or more?” Previous studies have found that depressive symptoms were more common in children whose parents were diagnosed with depressive symptoms [47,48]. This study tries to examine whether this association lasts over time.

### 2.3 Statistical analyses

Descriptive statistics, bivariate linear regression, and multivariate linear regression were conducted by SPSS. Descriptive statistics were calculated to describe the dependent depressive symptoms and to estimate the prevalence of geriatric depressive symptoms with the CESD-10 cut-off. Bivariate linear regression analyses were used to reveal the association between all independent variables and geriatric depressive symptoms. Furthermore, multivariate linear regression was used to examine the relationship between Childhood SES, being abused during childhood, and geriatric depressive symptom levels. In order to test the mediator role of being abused during childhood in the association between childhood SES and geriatric depressive symptoms, multiple mediation was conducted by the PROCESS macro for SPSS. This approach usually uses bias-corrected bootstrapping to measure multiple indirect effects [49,50]. The list-wise approach was used in all statistical analyses to deal with missing data, and all significance levels were set at P < 0.05.

## 3 Results

### 3.1 Descriptive analyses

Table 1 presented the prevalence of geriatric depressive symptoms, characteristics of childhood abuse experience, and SES, as well as socioeconomic features among respondents in this study. Among 8137 participants, around 36.29 % of them reported probable depressive symptoms, with a mean score of 8.75. About half of respondents (54.68 %) perceived their childhood SES as an average level, and nearly one in ten perceived their SES better than average. Regarding bullying behavior during childhood, most of the respondents reported no or few related experiences. Compared with parental violence and quarrels, fewer bullying behaviors occurred with siblings, schoolmates, and people within the same community. The percentages of people suffering from sibling abuse, school abuse, and community abuse were 15.72 %, 19.11 %, and 25.78 %, respectively. Among the respondents, 56.62 % were male, 75.86 % were from rural areas, and 92.43 % were of Han ethnicity. For individuals' marital status, 92.17 % had spouses, 2.42 % reported that it was not a voluntary marriage when they got married to their spouse, and 10.93 % stopped living with their spouse during adulthood. The mean age of participants was nearly 60, and 25.77 % of individuals perceived their health status as better, as well as 12 % and 29.98 % had diagnosable disability issues and chronic diseases respectively. In terms of parents’ health status, about 18 % and 11 % of the respondents said that when they were 17 years old, their mother or father had diagnosable depressive symptoms, respectively. And, 59.37 % of the subjects lost their father or mother during childhood and adulthood.

**Table 1:** Family SES in childhood, child abuse and socio-demographic characteristics among Chinese older adults. (N = 8137).

Variables		Frequency	Percent (%)
**Childhood SES**	**Family's financial situation**		
	A lot worse	1,637	20.12
	Somewhat worse	1,251	15.37
	same	4,449	54.68
	Somewhat better	716	8.80
	A lot better	84	1.03
**Child abuse**	**Parental violence**		
	Never	3,065	37.67
	Rarely	2,677	32.90
	Sometime	1,971	24.22
	Often	424	5.21
	**Sibling abuse**		
	Never	6,858	84.28
	Rarely	804	9.88
	Sometime	407	5.00
	Often	68	0.84
	**Community violence**		
	Never	6,039	74.22
	Rarely	1,128	13.86
	Sometime	732	9.00
	Often	238	2.92
	**School violence**		
	Never	6,582	80.89
	Rarely	884	10.86
	Sometime	525	6.45
	Often	146	1.79
	**Parental quarrel**		
	Never	3,603	44.28
	Not very often	2,682	32.96
	sometimes	1,468	18.04
	often	384	4.72
**Control variables**	**Gender**		
	Male	4,607	56.62
	Female	3,530	43.38
	**Hukou status**		
	Non-agricultural hukou	1,964	24.14
	Agricultural hukou	6,173	75.86
	**Marital status**		
	Have spouse	7,500	92.17
	Not have spouse	637	7.83
	**A voluntary marriage**		
	Yes	7,940	97.58
	No	197	2.42
	**Separation from spouse**		
	Yes	889	10.93
	No	7,248	89.07
	**Nationality**		
	Han nationality	7,521	92.43
	Minority	616	7.57
	**Religion**		
	Not Have	7,372	90.60
	Have	765	9.40
	**Self-report health status**		
	Better	2,097	25.77
	Poor	6,040	74.23
	**Disability issues**		
	Not have disabilities issues	7,160	87.99
	Have disabilities issues during childhood	58	0.71
	Have disabilities issues during adulthood	919	11.29
	**Chronic diseases**		
	Not have chronic diseases	6,023	74.02
	Have chronic diseases during childhood	10	0.12
	Have chronic diseases during adulthood	2,104	25.86
	**Parental death**		
	Not have	3,306	40.63
	Either father or mother died during childhood	682	8.38
	Either father or mother died during adulthood	4,149	50.99
	**Mother's mental health**		
	Not have depression	6,671	81.98
	Have depression	1,466	18.02
	**Father's mental health**		
	Not have depression	7,272	89.37
	Have depression	865	10.63
	**Depress**		
	Have	2,953	36.29
	Not have	5,184	63.71
	**Variables**	**Mean**	**Standard deviation**
	**Education years**	4.10	1.67
	**Income(Yuan/Year))**	4734.81	12902.34
	**Depress**	8.75	5.01

### 3.2 Linear regression analyses

Table 2 represents the results of the bivariate linear regression analyses, which revealed the relationship between independent variables and the level of depressive symptoms among the elderly. As shown, childhood SES and bullying behaviors were significantly associated with geriatric depressive symptoms. Poor childhood SES and exposure to more bullying behaviors were associated with considerably elevated scores. Individuals who were female, from rural areas, without spouses, without a voluntary marriage, separated from spouse, and belonged to minorities were at higher risk of depressive symptoms. Health status was associated strongly with depressive symptoms; poor self-reported health status, disability issues, and chronic diseases were related to higher depressive risk. Furthermore, parental mental health status and parental death were of great significance also. On the contrary, people with higher education and income would reduce the risk of developing depressive symptoms.

**Table 2:** Bivariate linear regression analysis of childhood SES, child abuse and depressive symptoms.

Variables	B	β	(95 % CI)
**Parental violence**	0.27	0.05***	(0.15,0.39)
**Sibling abuse**	0.60	0.07***	(0.41,0.79)
**Community violence**	0.56	0.09***	(0.42,0.70)
**School violence**	0.68	0.10***	(0.52,0.84)
**Parental quarrel**	0.44	0.08***	(0.32,0.56)
**Childhood SES**	0.58	0.11***	(0.47,0.70)
**Gender (Ref: Male)**			
Female	1.63	0.16***	(1.41,1.84)
**Hukou status (Ref: Non-agricultural hukou)**			
Agricultural hukou	0.74	0.06***	(0.49,0.99)
**Marital status (Ref: Have spouse)**			
Not have spouse	1.01	0.05***	(0.60,1.41)
**A voluntary marriage (Ref: Yes)**			
No	1.43	0.04***	(0.72,2.13)
**Separation from spouse (Ref: No)**			
Yes	0.75	0.05***	(0.40,1.10)
**Nationality (Ref: Han nationality)**			
Minority	0.48	0.03*	(0.07,0.89)
**Religion (Ref: Not have)**			
Have	0.26	0.01	(-0.12,0.63)
**Self-report health status (Ref: Better)**			
Poor	2.07	0.18***	(1.83,2.32)
**Disability issues (Ref: Not have disabilities issues)**			
Have disabilities issues during childhood	1.24	0.02	(-0.04,2.52)
Have disabilities issues during adulthood	2.12	0.13***	(1.78,2.46)
**Chronic diseases (Ref: Not have chronic diseases)**			
Have chronic diseases during childhood	5.13	0.04***	(2.05,8.21)
Have chronic diseases during adulthood	1.43	0.13***	(1.19,1.68)
**Parental death (Ref: Not have)**			
Either father or mother died during childhood	1.15	0.04***	(0.52,1.79)
Either father or mother died during adulthood	−0.02	−0.00	(-0.26,0.21)
**Mother's mental health (Ref: Not have depression)**			
Have depression	1.91	0.15***	(1.63,2.19)
**Father's mental health (Ref: Not have depression)**			
Have depression	2.04	0.13***	(1.69,2.39)
**Age**	−0.00	−0.00	(-0.01,0.01)
**Education years**	−0.37	−0.12***	(-0.44,-0.31)
**Income (Yuan/Year)**	−0.00	−0.10***	(-0.00,-0.00)

**Note**: *p < 0.05, ***p < 0.001; B: non-standardized Coefficient; β: standardized coefficient; CI: confidence interval.

Table 3 shows the results of multivariable linear regression, examining the relationship between childhood SES and level of depressive symptoms and the relationship between childhood SES, bullying behaviors, and level of depressive symptoms, respectively. After adjusting for socio-demographic characteristics and other confounding variables, childhood SES was significantly associated with depressive symptoms (β = 0.06, 95%CI 0.23–0.45). Childhood SES was still significantly correlated with geriatric depressive symptoms when bullying behaviors variables were included. Specifically, participants with disadvantaged SES during childhood reported higher depressive symptom levels (β = 0.05, 95%CI 0.17–0.40). Respondents with child abuse experiences, including sibling abuse (β = 0.02, 95%CI 0.02–0.40), community violence (β = 0.03, 95%CI 0.05–0.36), school violence (β = 0.04, 95%CI 0.10–0.46), parental quarrel (β = 0.03, 95%CI 0.06–0.31), were more likely to suffer from depressive symptoms, while parental violence was not associated significantly with depressive symptoms in the current study.

**Table 3:** Multivariable linear regression analysis of relationship between childhood SES, child abuse and depressive symptoms among Chinese adults. (N = 8137).

Variables	Model 1			Model 2		
	B	β	(95 % CI)	B	β	(95 % CI)
**Parental violence**				0.12	0.02	(-0.00,0.25)
**Sibling abuse**				0.21	0.02*	(0.02,0.40)
**Community violence**				0.20	0.03*	(0.05,0.36)
**School violence**				0.28	0.04**	(0.10,0.46)
**Parental quarrel**				0.19	0.03**	(0.06,0.31)
**Childhood SES**	0.34	0.06***	(0.23,0.45)	0.28	0.05***	(0.17,0.40)
**Gender (Ref: Male)**						
Female	1.39	0.14***	(1.17,1.61)	1.47	0.15***	(1.25,1.70)
**Hukou status (Ref: Non-agricultural hukou)**						
Agricultural hukou	0.21	0.02	(-0.06,0.48)	0.26	0.02	(-0.01,0.53)
**Marital status (Ref: Have spouse)**						
Not have spouse	0.63	0.03*	(0.11,1.15)	0.61	0.03*	(0.09,1.13)
**A voluntary marriage (Ref: Yes)**						
No	0.65	0.02	(-0.02,1.33)	0.50	0.02	(-0.18,1.18)
**Separation from spouse** (**Ref: No)**						
Yes	0.19	0.01	(-0.26,0.63)	0.17	0.01	(-0.28,0.62)
**Nationality (Ref: Han nationality)**						
Minority	0.47	0.02*	(0.07,0.86)	0.54	0.03**	(0.14,0.93)
**Religion (Ref: Not have)**						
Have	0.29	0.02	(-0.07,0.65)	0.31	0.02	(-0.05,0.67)
**Self-report health status (Ref: Better)**						
Poor	1.42	0.12***	(1.17,1.66)	1.36	0.12***	(1.12,1.60)
**Disability issues (Ref: Not have disabilities issues)**						
Have disabilities issues during childhood	0.49	0.01	(-0.74,1.71)	0.52	0.01	(-0.70,1.74)
Have disabilities issues during adulthood	1.64	0.10***	(1.31,1.97)	1.63	0.10***	(1.30,1.95)
**Chronic diseases (Ref: Not have chronic diseases)**						
Have chronic diseases during childhood	4.50	0.03**	(1.56,7.44)	4.66	0.03**	(1.73,7.59)
Have chronic diseases during adulthood	1.03	0.09***	(0.79,1.26)	0.99	0.09***	(0.75,1.23)
**Parental death (Ref: Not have)**						
Either father or mother died during childhood	0.91	0.03**	(0.31,1.51)	0.92	0.03**	(0.32,1.52)
Either father or mother died during adulthood	0.01	0.00	(-0.21,0.23)	0.02	0.00	(-0.21,0.24)
**Mother's mental health (Ref: Not have depression)**						
Have depression	0.98	0.08***	(0.64,1.32)	0.75	0.06***	(0.41,1.10)
**Father's mental health (Ref: Not have depression)**						
Have depression	0.81	0.05***	(0.39,1.24)	0.75	0.05***	(0.34,1.17)
**Age**	−0.02	−0.03*	(-0.03,-0.00)	−0.01	−0.01	(-0.02,0.01)
**Education years**	−0.12	−0.04***	(-0.20,-0.05)	−0.13	−0.04***	(-0.20,-0.06)
**Income(Yuan/Year)**	−0.00	−0.03**	(-0.00,-0.00)	−0.00	−0.03**	(-0.00,-0.00)

**Note**: *p < 0.05, **p < 0.01, ***p < 0.001; β: standardized coefficient; CI: confidence interval.

### 3.3 Examination on mediating effect

**Fig. 1:**
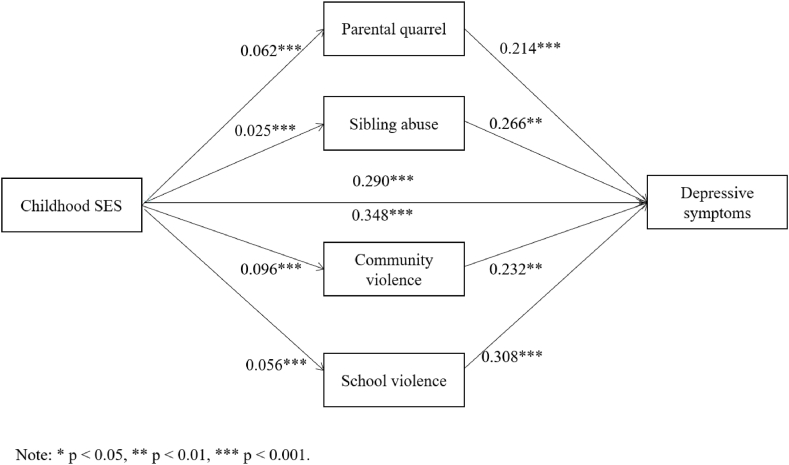
Child abuse as a mediator between childhood SES and depressive symptoms.

As shown in Table 4 and Fig. 1, we examined the mediator role of being abused during childhood in the association between childhood SES and depressive symptom levels. Meanwhile, outcomes of linear regression analyses enabled us to exclude parental violence from the multiple mediations. According to the total effect of childhood SES on depressive symptoms, childhood SES is associated significantly with geriatric depressive symptoms (B = 0.38, 95%CI 0.26–0.49). However, after adding bullying behaviors during childhood into the Model, the direct effect of childhood SES decreased to 0.32 (95%CI 0.20–0.43). While poor childhood SES related to higher bullying risk during childhood to some extent, the indirect effect of poor childhood SES to high geriatric depressive risk through community violence, sibling abuse, school violence, and parental quarrel were 0.02, 0.01, 0.02, and 0.01, respectively. To summarize, the results confirmed that being abused during childhood, including community violence, sibling abuse, school violence, and parental quarrel, partly mediated the relationship between childhood SES and geriatric depressive symptoms because the effect of childhood SES on depressive symptoms was still significant when bullying behaviors variables were included.

**Table 4:** Outcomes of mediation analyses in the relationship between childhood SES and depressive symptoms among Chinese older adults. (N = 8175).

	B	95 % CI
Total effect (c)		
Childhood SES → Depress	0.38***	(0.26, 0.49)
**Direct effect of childhood SES on depress (c’)**		
Childhood SES → Depress	0.32***	(0.20, 0.43)
Indirect effect of childhood SES on depress (a*b)		
Total Indirect effect	0.06	(0.04, 0.08)
Childhood SES → Community violence → Depress	0.02	(0.01, 0.04)
Childhood SES → School violence → Depress	0.02	(0.01, 0.03)
Childhood SES → Sibling abuse → Depress	0.01	(0.00, 0.01)
Childhood SES → Parental quarrel → Depress	0.01	(0.01, 0.02)

Note: Coefficients were adjusted for other variables in this table; B: Coefficient; CI: confidence interval.

## 4 Discussion

This study found that childhood SES was significantly and negatively associated with geriatric depressive symptoms. Furthermore, in line with the mediating relationship hypothesis, being abused during childhood, including community violence, sibling abuse, school violence, and parental quarrel, partly mediated the relationship between childhood SES and geriatric depressive symptoms.

This study's key findings need extensive discussion. Initially, our research confirmed the first controversy that early family socio-economic status was significantly and negatively associated with geriatric depressive symptoms. This association can be explained in several ways. Firstly, because all participants in this study were born before 1979, an era with prevalent poverty and hunger, individuals from low SES families faced more severe food shortages and deprivation of sanitation facilities, leading to life stresses directly and re-emerged depression in later life possibly. Similar associations were found in a prior study conducted in Japan, Iceland, and Mexico in the context of food shortage and lack of sanitation facilities [21]. Secondly, according to the theory of SES, People with high SES are more likely to have extensive social support and a sense of self-actualization, which act as protective factors to passive outcomes on mental health [51]. In addition, when we examined existing findings from a bullying perspective, the results suggested that participants with low childhood SES may be at higher risk of exposure to unwanted memories such as childhood abuse, and they are which have been shown to increase the probability of depressive symptoms further [52].

Furthermore, the findings support our hypothesis that child abuse, as a possible explanatory factor, slightly mediated the relationship between childhood SES and depressive symptom levels among the elderly. The pathway is that children from low SES families are possible to experience more childhood adversities, such as community violence, sibling abuse, school violence, parental quarrels and violence, which are associated with poor mental health outcomes well into late life. The potential mechanism is that children with lower SES tend to suffer more bullies and adversities from family and neighborhood (i.e. school, community) contexts [29]. This can be explained by that families with lower SES tend to be accompanied by more risk factors for child abuse, such as poor parenting skills and knowledge of child development, and parents' emotional illness [53,54]. Due to those complex factors, children are more likely to suffer from cognitive deficits, behavioral problems and emotional issues [55], which could predict peer abuse/violence to some extent [56], like school violence, sibling abuse and community violence. Then according to the Traumatic stress theory, the effect of these traumatic experiences would deteriorate people's psychological health status, even in their old age [57].

Particularly, we found that childhood SES was correlated with elderly depressive symptoms through parent quarrel. Previous studies also suggest that conflicts and arguments within the home were directly associated with the prevalence of depression [58]. A possible explanation could be attributed to Chinese culture. According to research, one in three Chinese people in China experienced physical abuse, one in five people experienced emotional abuse, and two in five people experienced neglect as children [59,60]. Child abuse and maltreatment were such strange concepts that it was difficult for the Chinese to realize and admit their existence [61], particularly in the family context. According to traditional Chinese culture, “beating is caring and scolding is loving.” Before 1979, when our participants were young children, this firmly ingrained traditional idea had a significant impact on them. Even now, there are still many Chinese parents who hold that idea. This prevalent perception passes across the generation gap, and even children themselves accept the abusive behaviors from their parents as necessary discipline. In fact, it has not been easy to distinguish child abuse from harsh child discipline among Chinese families [62]. In addition, community and school violence have been defined as the exposure to violence and violence-related events occurring around the home and may involve physical as well as mental harm, like depression [63,64]. One possible explanation is that neighborhood disadvantage could shape the development of impulsivity and provide the enabling environment for violence and aggressive behaviors [65], and bullying was recognized as a special form of aggressive behavior [56]. All of these together suggest that low SES in the early family may lead to childhood abuse and bullying, which might then worsen depressive symptoms in the elderly.

Finally, our findings indicated that geriatric depressive symptom level was more severe among females and those without spouses, which is in accordance with previous studies [66,67]. Considering the huge gap in economic development between rural and urban areas, a significant association between residence and depression could be explained by financial issues and public resource delivery to some extent [16]. Meanwhile, individuals with higher education were more likely to have life skills and resources needed for promoting health and thus have a lower risk of depressive symptoms as revealed in a previous study [68]. Aging has been considered to have an important detrimental effect on mental health and the prevalence of depression could increase with age due to economic and physical dependency, loss of the company, and self-esteem [69,70]. However, no significant relationship between age and depression symptoms was found in our study. In addition, the findings showed that income was a protective factor for mental health status in the elderly. A study by Areán and his colleagues also showed similar results, indicating that elderly persons with low incomes are exposed to several risk factors for depression, such as poor nutrition, poor physical health, and poor mental health services [71]. Furthermore, several existing studies support that parental depressive symptoms increase the probability of depressive symptoms among their children; parents with mental disorders are associated with less optimal parenting, higher problem behavior, lower social competence and less adaptive emotion regulation strategies, these factors are found to be related to children's mental disorders [72]. A previous study has also found that minorities were more susceptible to depressive symptoms, possibly due to discrimination within society or cultural differences [73]. However, the current study found no significant association between nationality and geriatric depressive symptoms. A possible reason for this contradiction could be that, among our cohort, the societal and cultural differences were not as powerful as in Western societies. Nevertheless, further investigation of this association is called for.

Nowadays, there has been an increase in child abuse cases worldwide since the COVID-19 epidemic [74]. The COVID-19 lockdown methods limit children's access to basic services like food and healthcare as well as various types of social support. As a result, children are more likely to experience sexual, physical, and emotional abuse as well as neglect [75]. Additionally, with the pandemic's psychosocial effects and the subsequent economic collapse, parents and citizens became stressed and aggressive due to a lack of social support, increasing the likelihood that children will encounter violence at home and in the community [76].

This study recommends that a developmental perspective is needed to understand childhood SES across the life course. Implementing policies against child poverty is helpful to improve individuals' mental health throughout their lifespan. Close attention should be paid to bullying behaviors toward children. Targeted intervention services should be delivered to those being abused during childhood, particularly in families with low SES. Preventative measures in families, schools, and communities should be established to alleviate negative effects on mental health outcomes. Moreover, individuals who were female, from rural areas, without spouses, without a voluntary marriage, separated from spouse and with lower education and incomes were at higher risk of depressive symptoms and should receive more attention to prevent suffering from depressive symptoms.

Some limitations should be mentioned in this study. Firstly, measures of SES and bullying experiences during childhood are based on retrospective self-reporting, which may lead to recall bias, particularly when the events occurred in very early life stages. Those with depressive symptoms may recall these negative events more than those without. However, previous studies with longitudinal follow-up of adults whose childhood abuse was well documented found that retrospective reports are likely to underestimate rather than overestimate the prevalence of abuse [77]. Future research could extend single-source methods to obtain more objective information, thus avoiding memory or self-perception bias. Secondly, the CESD is a self-report instrument rather than a clinical diagnostic measure, which may be over- or under-reporting of symptoms [8]. Future work should adopt objective rather than subjective measures aimed at securing the validity of the results. Thirdly, not all types of child abuse were measured in the analysis, although potential types of child abuse in early childhood environments were included. Due to limited resources, some potential confounding factors such as occupation, exercise frequency, relationship with children, and so on have not been considered and controlled. Furthermore, the relationship and pathways between childhood SES and depressive symptoms are complex, and other potential personal and social resources that may buffer relationships between childhood SES and depressive symptoms among Chinese older adults should also be taken into account in the future.

## 5 Conclusions

This study aims to explore the mediating effect of being abused during childhood within the association between childhood SES and geriatric depressive symptoms. The main conclusions are as follows: (1) This study confirmed a significant negative correlation between lower childhood socioeconomic status and higher levels of depressive symptoms. (2) Regarding the mediating effect, if the individuals have lower levels of SES, this may make them more prone to suffer childhood abuse containing community violence, sibling abuse, school violence, and parental quarrel, which can lead to higher levels of geriatric depressive symptoms.

Depression among Chinese older adults is a significant public health issue in need of more attention. This study highlights the importance of inquiring about childhood adversity, including childhood SES and bullying behaviors, and the need to promote childhood social welfare as well as psychological well-being within the elderly population. Our findings enrich our understanding of how childhood SES can increase the risk of geriatric depressive symptoms through childhood abuse and fill a gap in the research on the relationship between childhood SES and geriatric depressive symptoms in the context of Chinese society. In further study, it's important to consider other personal and social resources that could act as a buffer between older Chinese individuals' depressive symptoms and their childhood SES.

## Data availability statement

Data will be made available on request.

## Ethics statement

This survey has been approved by the Research Ethics Committees of 10.13039/501100007937Peking University Medical Center (IRB00001052-11015), and that it conforms to the provisions of the Declaration of Helsinki (as revised in Tokyo 2004). All the respondents were required to give informed consent before participation.

## Funding

This work was supported by the National Social Science Fund of China (Number: 22VJXT010).

## CRediT authorship contribution statement

**Chengcheng Liu:** Writing – original draft. **Mingyu Zhang:** Writing – review & editing, Data curation. **Chongyue Ma:** Writing – review & editing. **Mingqi Fu:** Writing – review & editing. **Jing Guo:** Writing – review & editing, Supervision. **Cheng Zhen:** Writing – review & editing, Conceptualization. **Bo Zhang:** Writing – review & editing.

## Declaration of competing interest

The authors declare that they have no known competing financial interests or personal relationships that could have appeared to influence the work reported in this paper.
