# Supplementary material for: Childhood abuse as a mediator of the relationship between early family socio-economic status and geriatric depression: A population-based study in China
Source: Heliyon. 2023 Nov 7;9(11):e22021. doi: 10.1016/j.heliyon.2023.e22021 (PMC10682629; doi:10.1016/j.heliyon.2023.e22021)
Supplement: Multimedia component 1 [file mmc1.pdf]

<u>**Form to confirm authorship changes for *Heliyon***</u>

This form must be ***signed by all authors*** when there is a change in authorship which includes changes to any of the following items: author name(s), order of the authors, the corresponding author(s), the addition of authors, the removal of authors and changes in affiliation.

By personally signing this note, **all** authors confirm that: I) the changes are in accordance with their scientific contribution, II) they agree with all the changes and III) confirm that the authorship list conforms to the authorship criteria outlined on [*Heliyon*'s ethics page]. IV) it is the responsibility of the corresponding author to get the signature from all co-authors accepting the change. In case of any ethic violation/malpractice in the signature, the corresponding author is accountable. The completed form should be returned along with the final/revised manuscript to proceed further with the manuscript. Manuscripts for which incomplete forms have been submitted will be rejected within 5 working days.

Any disputes on the authorship list and contributions need to be resolved by the involved scientists and *Heliyon* will only proceed with the evaluation of the manuscript once we receive confirmation, through this form, that such an agreement between the authors has been reached.

***Heliyon*** **will not accept changes to the authorship list in the late stages of the editorial process (when a paper is in Accept in Principle stage, acceptance or after publication)**

Manuscript number: HELIYON-D-23-13368R1

Article title: Childhood Abuse as a Mediator of the Relationship between Early Family Socio-Economic Status and Geriatric Depression: A Population-Based Study in China

Complete new author list: Chengcheng Liu, Mingyu Zhang, Chongyue Ma, Mingqi Fu, Jing Guo, Cheng Zhen, Bo Zhang #Co-first author

Date: 2023.11.01

| # | First name | Last name | Dept. & Institution name | Institutional email address | Order change (Y/N) | Addition / Deletion | Change in Author name (Y/N) | Affiliation Change (Y/N) | Reason for the change | Signature |
|---|---|---|---|---|---|---|---|---|---|---|
| 1 | Cheng cheng | Liu | The School of Social Development and Public Policy, Beijing Normal University, Beijing 100875, PR China | zhencheng@bjmu.edu.cn | Y | | N | N | CL & MZ drafted the manuscript and analyzed the data. | Chengcheng Liu |

| | | | | | | | | | | |
|---|---|---|---|---|---|---|---|---|---|---|
| 2 | Mingyu | Zhang | School of Public Health, Peking University, Beijing 100191, PR China | zhangmingyu189@163.com | Y | Addition | N | N | CL & MZ drafted the manuscript and analyzed the data. | Mingyu Zhang |
| 3 | Chongyue | Ma | School of Accounting, Henan University of Economics and Law | 2680475501@qq.com | Y | Addition | N | N | MF, BZ, ZZ, CM, JG revised the manuscript. | Chongyue ma |
| 4 | Mingqi | Fu | School of Public Management, Central south University, Wuhan 430079, PR China | Hdwyandot@163.com | Y | | N | N | MF, BZ, ZZ, CM, JG revised the manuscript. | Mingqi Fu |
| 5 | Jing | Guo | School of Public Health, Peking University, Beijing 100191, PR China | jing624218@163.com | Y | | N | N | JG & ZZ design the study. | Jing Guo |
| 6 | Cheng | Zhen | School of Health Humanities, Peking University Health Science Center | zhencheng@bjmu.edu.cn | Y | Addition | N | N | JG & ZZ design the study. | Cheng Zhen |
| 7 | Bo | Zhang | Children's Hospital and Harvard Medical School | Bo.Zhang@childrens.harvard.edu | Y | | N | N | MF, BZ, ZZ, CM, JG revised the manuscript. | Bo Zhang |
